# Nutrition education incorporation into mainstream primary school curriculum in Ghana: Stakeholders’ sources of nutrition information and perceived barriers

**DOI:** 10.1371/journal.pone.0262359

**Published:** 2022-01-06

**Authors:** Esi Quaidoo, Agartha Ohemeng, Mawuli K. Kushitor, Janet Antwi

**Affiliations:** 1 Department of Nutrition, School of Public Health and Health Sciences, University of Massachusetts, Amherst, Massachusetts, United States of America; 2 Department of Nutrition and Food Science, University of Ghana, Accra, Ghana; 3 Department of Health Policy Planning and Management, School of Public Health, University of Health and Allied Sciences, Ho, Volta Region, Ghana; 4 Department of Agriculture, Nutrition and Human Ecology, Prairie View A&M University, Prairie View, Texas, United States of America; University of Washington, UNITED STATES

## Abstract

**Introduction:**

Nutrition literacy has been cited as a crucial life skill. Nutrition education as a primary school subject has been treated inconsequentially when compared to other subjects. We investigated an aspect of the current state of nutrition education in Ghana by engaging stakeholders about their sources of nutrition information and the perceived barriers in implementing nutrition education in mainstream primary schools.

**Methods:**

Three hundred and fifty one (351) primary school children, 121 homebased caregivers, six schoolteachers, two headteachers, two Ghana Education Service (GES) officials, and six school cooks were involved in the study. Surveys were used to collect data on nutrition information acquisition behaviors and to record perceived barriers. Key Informant Interviews were conducted among GES officials, headteachers, schoolteachers and school cooks, while Focus Group Discussions were used among homebased caregivers and children to gather qualitative information.

**Results:**

Only 36.3% of the primary school children had heard about nutrition, and 71% of those got nutrition information from their family members. About 70% of homebased caregivers had heard or seen nutrition messages, and their source of nutrition information was predominantly traditional media. Schoolteachers mostly received their nutrition information from non-governmental organizations and the Internet, while most of the school cooks stated their main source of nutrition information was hospital visits. Perceived barriers included schoolteachers’ knowledge insufficiency, and lack of resources to adequately deliver nutrition education. Lack of a clear policy appeared to be an additional barrier.

**Conclusion:**

The barriers to the implementation of nutrition education in the mainstream curriculum at the primary school level that were identified in this study can be resolved by: providing schoolteachers with learning opportunities and adequate nutrition education resources for practical delivery, having specific national policy framework, and including family members and school cooks in the nutrition education knowledge and information dissemination process.

## Introduction

Families play a major role in the genesis and expansion of nutrition behaviors due to the responsibility of feeding a child [1]. The family unit sets the basis for food norms to be established by acting as a role model and encouraging certain behaviors while rewarding or limiting others [1]. As children start formal schooling, extrafamilial influences progressively become more important references for nutritional behaviors [2]. As such, the school environment presents a good opportunity to address broad level systemic challenges of child nutrition [3]. At this point, not only family members are influences but friends, peers and social models become additional impacts on children’s nutrition behaviors [2]. Research suggests that a lack of nutrition knowledge among children and their caregivers affects nutrition behaviors, and this has been linked to pediatric weight gain [4–6]. Other research links the active engagement of actors (e.g., schoolteachers) within the school environment in the acquisition of nutrition knowledge with a lower risk of children developing childhood obesity and/or experiencing undernutrition [2, 7–9].

The double burden of malnutrition is reported to be increasingly occurring among children of school going age especially in developing countries [10]. The Global Action Plan for non-communicable diseases along with the Rome Declaration on Nutrition and Framework for Action have all called for an intensification of action within the school environment to aid children maintain healthy nutrition statuses [11]. The World Health Organization has specifically called for the regulation of nutrition within the school environment to end childhood obesity [12]. These major international policies have trickled down into national educational policies with the aim of developing Nutrition Education (NE) within the school system. While these major policies have often generated a lot of interest among stakeholders, there is sufficient evidence to show that NE policies have not produced expected results in the school environment, due to varied reasons including underfunded and under-resourced NE programs in schools [11, 13]. In Ghana, for example, a comparison of the Food and Agriculture Organization (FAO) Guidelines for Nutrition Education in Primary Schools for Developing Countries and Ghana’s Primary School Teaching syllabus shows shortfalls in the curriculum [14, 15]: the policy brief on Primary School Teaching in Ghana does not explicitly mention a NE policy to guide actors within the school environment [15].

The International Food Policy Research Institute’s 2016 Global Food Policy Report has subsequently called for research into the enablers and barriers of nutrition education implementation in schools [16]. Research is however limited on the extent to which different kinds of barriers and enablers may affect NE in different school environments. Only a few studies offer an African perspective on the current state of NE in primary schools. Furthermore, studies from the Ghanaian perspective that describe the barriers involved in the integration of nutrition as a standalone subject into the mainstream primary school curricula are lacking. Thus, this study explored the current state of NE by first assessing nutrition information acquisition behaviors among children of school going age, homebased caregivers, schoolteachers and school cooks in Ghana. Secondly, barriers in the implementation and maintenance of NE in primary schools were examined.

## Materials and methods

### Study design

The analyses provided in this paper forms an aspect of a parent study [17] that focused on a sample of primary school children, their homebased caregivers, and schoolteachers. The parent study’s general aim was to determine the impact of a six-week nutrition education intervention on the nutrition knowledge, attitude, and practices of school-age children in Ghana [17]. Available quantitative and qualitative data were analysed to examine the nutrition information acquisition behaviours of study participants as well as shed light on barriers in the incorporation of nutrition education into mainstream primary schools in Ghana. The quantitative section of the data was gathered using cross-sectional surveys whereas the qualitative section of the data presented was obtained via Key Informant Interviews (KII) and Focus Group Discussions (FGD) with the aim of making observations at different levels. Aside FGD, KIIs were deemed necessary to use because of the sensitivity of some questions posed. Moreover, such an approach helped us to meet interviewees at their convenience, and it facilitated generation of information by aiding them to express recollections.

### Study area

The parent study was conducted in Dzorwulu (an urban enclave), and Brepaw Upper and Fefe (a rural area) from June 2018 through December 2018. Dzorwulu is an affluent area in Accra with well-planned residences in the Ayawaso West Municipal District, of the Greater Accra Region of Ghana. Whereas, Brepaw Upper and Fefe are two villages in the Aseseswa sub-district of the Upper Manya Krobo district in the Eastern Region of Ghana [18]. The schools we selected in the urban area had spacious and clean compounds. There were school feeding programs provided and food vendors were present. The schools catered to mostly lower middle class families. Both schools selected in the rural setting were also spacious and clean but lacked more amenities (e.g. running water) than their urban counterparts. School feeding programs were present but underfunded. The schools catered mostly to lower class families.

### Study population and sampling

The study population, herein referred to as stakeholders consisted of school-going children aged 6 to 12 years in grades 1 to 6 and their primary homebased caregivers, schoolteachers, headteachers, Ghana Education Service (GES) officials, and school cooks. In each study area, two public primary schools were conveniently selected to be included in the study after obtaining permission from the respective district directorates of the Ghana Education Service. A total of four hundred school-going children were randomly sampled and interviewed but three hundred and fifty-one completed the survey that the current paper is reporting on. Twelve of the school-going children also participated in FGD. One hundred and twenty-one homebased caregivers participated in the survey and twelve in FGD. Schoolteachers, headteachers, and school cooks, and GES officials, were purposively selected as actors within the school system/community who were directly in charge of managing the school community, directly handling food, and as policy implementers, respectively. Six each of schoolteachers and school cooks, two headteachers, and two GES officials were interviewed using KII.

This study was conducted according to the guidelines laid down in the Declaration of Helsinki and all procedures involving research study participants were approved by the College of Basic and Applied Sciences, University of Ghana Institutional Review Board (IRB, {study # ECBAS 029/17-18}) and State University of New York at Oneonta IRB (study # 529). Approvals were sought from the Accra Metropolitan Assembly directorates and Upper Manya Krobo District of the Ghana Education Service and school principals of the participating schools. Voluntary informed consent was obtained from all adult participants before the study was conducted. Homebased caregivers provided consent for themselves and their children’s participation in this study. In addition, children seven years and older completed assent forms to participate in the study.

### Data collection

The primary school children and homebased caregivers were interviewed using a semi-structured questionnaire and twelve each were engaged separately in four FGD (i.e. three children per session, and three caregivers per session) due to a saturation of information. We conducted KII to collect in-depth information on nutrition acquisition and perceived barriers from schoolteachers, headteachers, and school cooks, and GES officials. All FGDs and KIIs were audio-recorded using a recording device. The KII were thirty to sixty minutes long while FGD were about thirty to forty-five minutes long. The interview guides employed in this study are included as Supporting Information file. The quantitative questionnaires were designed based on researchers’ prior knowledge of the study population and on previously published instruments [19–22]. The assessment tools and procedures were pre-tested in a similar population, at a different location, before the tools were implemented in the main study.

#### Nutrition information acquisition

School-age children were asked whether they knew what nutrition was. If a child responded “no”, research assistants explained nutrition to the child and asked if they had encountered any nutrition or nutrition-related messages and what source the message came from. Questions were included in the homebased caregivers’ surveys on whether they had encountered nutrition messages and the possible reasons why they may not have encountered nutrition messages thus far. Focus group discussions were organized for homebased caregivers and children to gain a better understanding of their interactions with nutrition information. Questions for the schoolteachers and school cooks included whether they talked about nutrition in class, whether they gave examples of healthy balanced meals in class and whether they interact with children in an attempt to encourage them to participate in nutrition related activities (i.e. if any are done in school). To gain further insight into where schoolteachers and school cooks accessed nutrition information, KII were conducted.

#### Perceived barriers

Data on perceived barriers in delivering and sustaining nutrition education at the primary school level was obtained via KIIs. Teachers were also asked to provide information on the drawbacks they may have faced in delivering nutrition education to their pupils. GES officials and headteachers were also interviewed on policies.

### Analyses

Quantitative data underwent descriptive analyses and was presented as counts and percentages using SPSS Version 23.0. The qualitative data in the study employed thematic content analytical approach. All transcripts were appropriately labelled after the interviews. Professional transcribers with competences in the languages of interviewees were further trained on the level of transcription required for this analysis. That is, a coauthor and data analyst (MKK), and the lead researcher and coauthor (JA) selected and trained specific fieldwork assistants on transcribing recordings to the level of detail of project objectives, local context, and interview guides for representation of the phenomenon under study. All interviews were transcribed verbatim. Transcripts were read thoroughly by the research team to familiarize with the text. To ensure inter coder reliability, the researchers had extensive deliberations on what should constitute a code and constantly resolved discrepancies throughout the coding process. A code was used based on the ideas and overall groupings of nutrition information acquisition, and perceived barriers in the interview guides. The researchers proceeded with the coding process which involved going through respondent transcripts, and transcripts were coded by labelling the text with codes (e.g. Source of Nutrition Information, and Definition of Nutrition) demonstrating similar concepts that emerged as themes. After a thorough process of coding one transcript, the same process was applied in coding the rest of the transcripts. Through the process of constant comparison, similar ideas occurring in the transcripts were labelled the same way to ensure the uniformity of the codes and their representations across the entire data corpus.

## Results

A total of 351 school-age children, 121 homebased caregivers, six schoolteachers, two headteachers, two GES officials and six school cooks were interviewed. All of the school cooks were female, whereas the headteachers and GES officials were all male between 45–55 years old. Majority of the homebased caregivers were female, had a junior high school education, residents in urban areas and a parent of at least one of the children that were interviewed (Table 1).

**Table 1:** Sociodemographic characteristics of study participants.

Sociodemographic Characteristic	Children	Homebased Caregivers	Teachers
**Gender** n (%)			
Male	178 (50.7)	32 (26.4)	3 (50.0)
Female	173 (49.3)	89 (73.6)	3 (50.0)
Total	351 (100)	121 (100)	6 (100)
**Age, years** Mean (±SD)	9.6 (1.8)	38.53 (10.83)	33.0 (3.8)
**Area of Residence** n (%)			
Urban setting	198 (56.4)	75 (62.0)	3 (50.0)
Rural setting	153 (43.6)	46 (38.0)	3 (50.0)
Total	351 (100)	121 (100)	6 (100)
**Highest Level of Education** n (%)			
No Formal Education		23 (19.0)	0 (0)
Primary School Education		16 (13.2)	0 (0)
Junior High School Education		43 (35.5)	0 (0)
Senior High School Education		30 (24.8)	0 (0)
Vocational School Education		5 (4.1)	2 (33.3)
University Education		4 (3.3)	4 (66.7)
Total		121 (100)	6 (100)
**Relationship to Pupil** n (%)			
Parent		99 (81.8)	0 (0)
Relative		19 (15.7)	0 (0)
Other*		3 (2.5)	6 (100)
Total		121 (100)	6 (100)
**Pupil lives with** n (%)			
Both parents	245 (69.8)		
Single parent	72 (20.5)		
Relative	34 (9.7)		
Total	351 (100)		

Shaded areas indicate information is not applicable.

*Other includes teacher at school and family friend.

### Nutrition information acquisition

#### School-age children

Three hundred and fifty-one primary school-aged children were interviewed and only 127 (36.3%) had heard of nutrition. Of these 127 children, most reported acquiring nutrition information from family members, followed by lessons taught in class (Table 2).

**Table 2:** Sources of nutrition information of parents and children.

	n(%)
School-Age Children’s Sources of Nutrition Information* N = 127	
Family members	90 (71.0)
Lessons in class	87 (68.5)
Television	55 (43.3)
Textbooks	29 (22.8)
Friends	19 (14.9)
Radio	18 (14.2)
Newspapers	4 (3.1)
Caregivers’ Sources of Nutrition Information* N = 85	
Traditional Media	36 (41.2)
Community Health Workers	21 (24.7)
Other Sources	18 (21.1)
NGOs	5 (6.0)
Family members and peers	5 (6.0)
School Feeding Program	1 (1.1)

*Study participants had multiple choices and were encouraged to pick all the sources that applied to them.

#### Homebased caregivers

Of the 121 homebased caregivers who filled out the survey, 85 (70%) had ever heard or seen a nutrition education message and most of them (84 out of 85 caregivers i.e. 98.8%) had found the nutrition information beneficial. A broad report of homebased caregivers’ sources of nutrition information is shown in Table 2.

**Fig 1:**
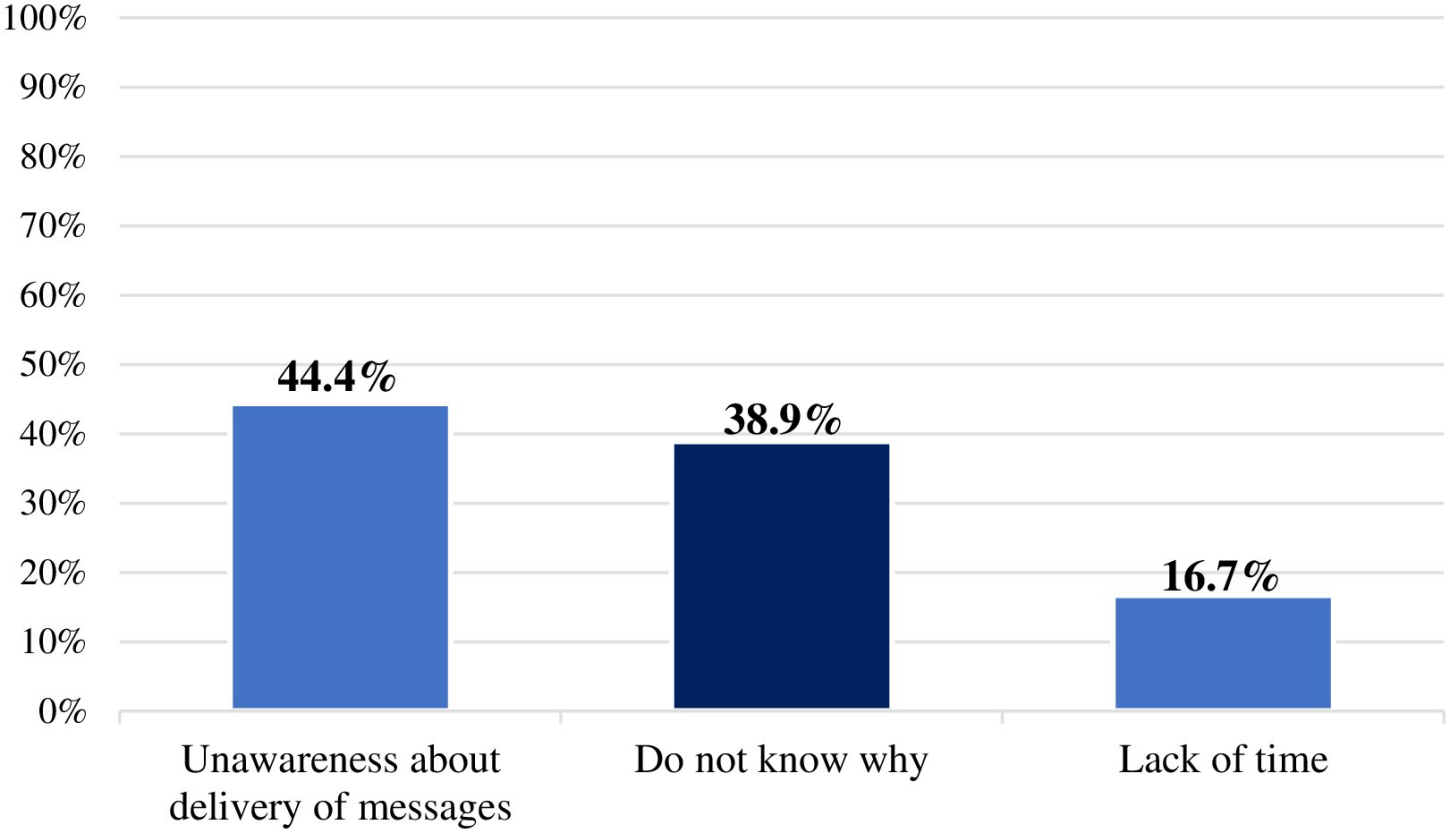
Self-reported reasons why caregivers had not heard or seen nutrition messages.

Of the 85 homebased caregivers who had ever seen or heard a nutrition education message, 36 (41.2%) stated traditional media as their main source of nutrition information. The 36 caregivers who reported never seeing or hearing a nutrition education message before were asked why that was the case, 16 (44.4%) reported that they were unaware about the delivery of nutrition information messages (Fig 1).

In FGD, of the themes that emerged, television and radio sources dominated the sources of nutrition information expressed by homebased caregivers (Table 3). External sources of information referred to information provided to the school or community environment by outside agents such as NGOs and other related institutions who are not necessarily mandated to carry out these functions. Institutions mentioned were PLAN Ghana, The University of Ghana and Lower Manya Krobo Rural Bank. Specifically, “*Telenurse*” was mentioned as homebased caregivers’ most authoritative source of nutrition information. Radio programs were also another important source of information.

**Table 3:** Summary of identified themes from key informant interviews & FGD.

Codes	Identified Themes
**Sources of Nutrition Information**	
Children	Parents, health professionals’ visits to school, books, electronic media (i.e. radio and television programs).
Homebased Caregivers	Religious bodies, television, radio, friends and social media, agricultural officers.
Teachers	External sources, the internet, social media, books, intuition, personal experience and popular domain.
School Cooks	Hospital visits, church, radio, television and the open market.
**The Definition of Nutrition**	
Homebased Caregivers	Eating fruits, eating vegetables, growth and health.
Teachers	Eating fruits, eating vegetables, brain development, growth, strength, health and holistic development.
School Cooks	Eating fruits, eating vegetables, good meal preparation and growth.

Two nutrition information sources that were absent in key informant interviews but present in the FGDs were the church and agricultural officers.

The results of KII provided further details in addressing the project objectives.

#### Teachers

Teachers interviewed identified receiving their nutrition information mostly from external sources while others received their information from the internet. They, however, did not mention the specific sources from which they obtained their information.

“Another source is the Upper Manya rural bank, they have a unit that is responsible for that, they are especially concerned about the girl child education, and hence education is focused mainly on the girl child and not the entire community. They have a body in this school. Another source is from people like you, who come into the community for their projects. PLAN Ghana also comes in sometimes to teach the teachers, students, and the community. As for friends, because I don’t live in the community, I can’t talk about how friends educate them. Normally, when the bodies are in, they teach we the teacher, the students and the community…”

*Key Informant Interview #5*

Several definitions of nutrition were presented during key informant interviews. One teacher presented a concept that was close to the classic definition of nutrition.

“In simple terms, nutrition is a means of introducing vital or important diet into our system by taking in food. All these things are included in our food and water, the important, good and healthy ones…”

*Key Informant Interview #2*

#### School cooks

Information from the hospital was the most important source in terms of frequency for the food service personnel that were interviewed.

“When you go to the hospital, they encourage you to eat fruits. Sometimes when you eat fruits, it’s good for your health. Sometimes, when you’re having stomach aches, they can ask you to take oranges. Sometimes you’d be asked to eat certain fruits in order to have a smooth skin…”

*Key Informant Interview #6*

In responding to the question about what exactly nutrition was, several definitions were put forth.

“Eating food that will give you strength and help in the development of the children’s brain…”

*Key Informant Interview #6*

“What will make food healthy is first to prepare it well. It must also contain leafy vegetables. Garden eggs are also in season now. So those are the things which are healthy. After eating that you can also take some fruits in addition to get good health…”

*Key Informant Interview #7*

### Perceived barriers

#### Knowledge insufficiency

**Fig 2:**
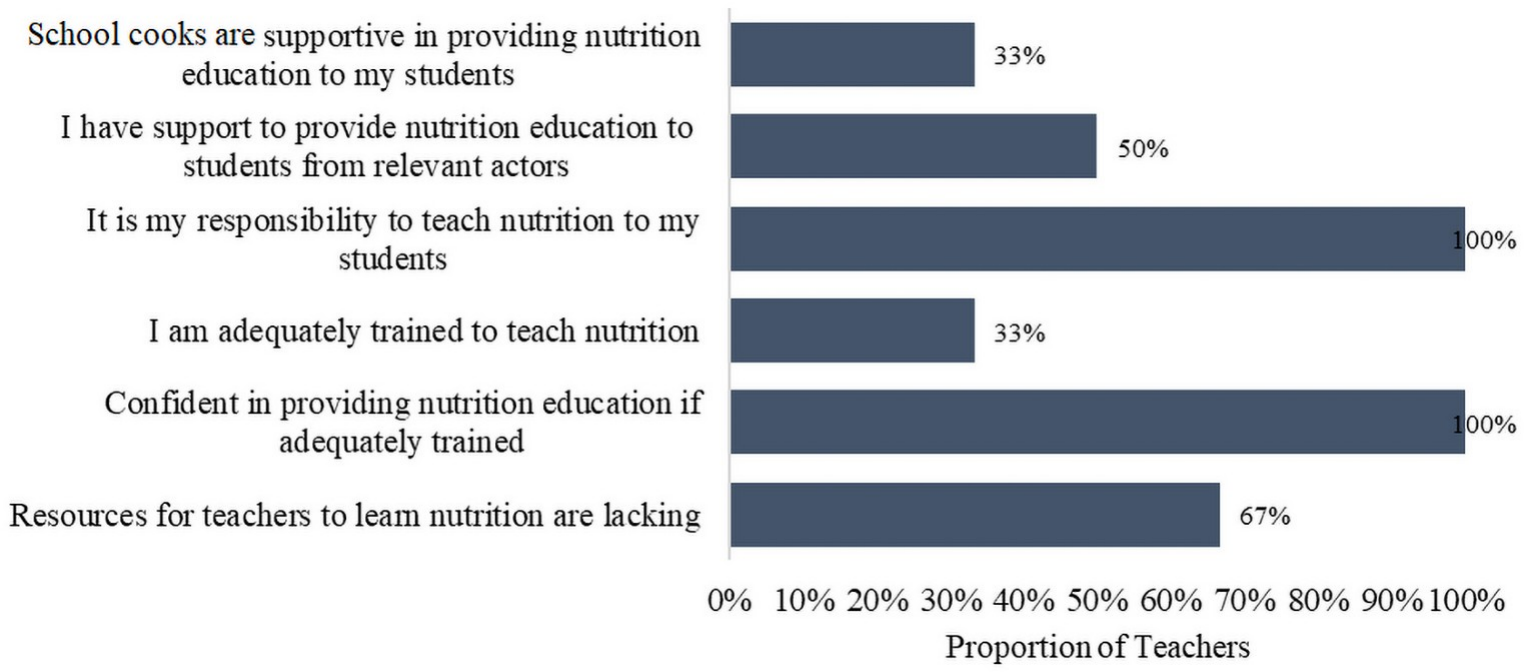
Teachers’ feedback on providing nutrition education at their schools (n = 6).

Teachers that were interviewed felt they were without the requisite skill to handle NE, leaving them at a disadvantage to adequately impart the children with nutrition knowledge. Only 33% felt adequately trained to teach pupils nutrition (Fig 2).

A key informant teacher indicated that delivery of nutrition education was not incorporated into her training during her time at Teachers’ Training College.

“During my school days I never paid attention to any nutrition book. It’s not part of general coursework. I learned social studies so I have never read any nutrition book that I would …. I would tell you the truth…”

*Key Informant Interview #3*

Teachers admitted limitations in their knowledge and how that could possibly affect their attitudes and behaviors towards the subject of nutrition.

Interviewer: What materials do you use for teaching Nutrition in the schools?

Respondent: We have a book that was given to us when we went for the workshop. It’s just a book. I don’t know if it’s here. It’s a book.

Interviewer: Is it adequate?

Respondent: No!

Interviewer: What practical skills do you offer that contribute to nutrition practice among the children?

Respondent: We don’t have any practical skills….*Key Informant Interview #4*

All the school cooks, however, noted that their level of nutrition knowledge was sufficient for managing food at the school canteens:

“If I should say no, the person who cooks for the school should have a kitchen, then you’d ask me ‘where’s my certificate before I’m cooking for the school?’ So for that, everyone and the kind of work they do…”

[*The school cook is essentially saying that although she does not have adequate knowledge on nutrition, she could not admit that to authorities because she does not have the certification for what she is doing and does not want to implicate herself] Key Informant Interview #7*

#### Lack of resources for practical delivery and active participation in nutrition education

This is probably because resources were generally limited especially within the public-school space. These limited resources were channeled into areas where the GES felt it was needed most as reported by a Senior GES official.

*“… a lot has been achieved but we are far from the destination*. *The facilities are not there and equipment are very expensive*. *If you want quality equipment*, *it is not easy to come by and funding is also a problem because when you come down to the schools for instance*, *everything in the school is run by capitation and capitation is sub divided*. *Some for sports*, *academics*, *repairs and others…*”

*Key Informant Interview #1*

Although head teachers mentioned that the capitation grant (this is a type of fund provided by the Ministry of Education to all public schools in Ghana) makes some allocation of funds, they were not directly accessible for nutrition and physical education. They may have to subsume those budgets under something else. Other critical challenges noted were a lack of study materials with which to teach the children nutrition. Teachers lamented the absence of key materials including models and kits to aid teaching and engaging their pupils in practical delivery of Nutrition and active participation of students. This made nutrition education delivery, if done, a largely theoretical approach.

#### Nonexistence of an explicitly stated policy on nutrition education

Teachers mentioned that the incorporation of NE was not explicit in the curricula; teachers were expected to give tidbits on nutrition whilst teaching other subjects to students. The focus was on “health education” which emphasized personal hygiene and not promoting nutrition behaviors.

“Yes! It’s known as inclusive education. It is done as a form of inclusive education; chipping in other relevant topics when teaching. For instance, you have to chip in HIV alert and issues on adolescence when you’re teaching a subject like Science…”

*Key Informant Interview #3*

A Senior GES official agreed that there was a NE policy, but he could not state what it was. He noted that the NE policy was inconspicuous, and that the policy has long been recognized by all the key stakeholders as essential. The major challenge has been on how to translate the policy through the various structures of GES into the school environment.

“*We just have to see the policy makers*, *basically GES we implement policies*. *The Ministry formulates polices and GES implements them*. *As I speak now*, *I think the curriculum is now being worked on*. *There is a team working on it*. *It is a matter of getting it out there… I just want to say that this must be spearheaded*. *It is there in nitty gritty in our curriculum*, *but it must be looked at and pushed up*. *All that we are talking about are there*, *but we need to push it up so that it becomes a policy for people to stress on and do and definitely it would help us a lot*. *I think if it gets to the ministry who are the policy formulators*, *they would put it out there and we would implement it*. *I believe anyone you approach with this would say this is a necessity*. *It is embedded there but it is not being pushed so it would be in the limelight…*”

*Key Informant Interview #1*

## Discussion

This study sought to assess stakeholders’ (i.e. the various units invested in delivering and receiving nutrition information) nutrition information acquisition behaviors and the perceived barriers involved in the incorporation of nutrition education into the mainstream Ghanaian primary school curriculum. The quality of Nutrition Education (NE) related activities is a function of the quality and level of information received [23], as such, all study respondents were asked about their nutrition information acquisition behaviors. A low number of the interviewed school-age children knew what nutrition was to begin with. This observation is similar to the study by Mamba, Napoles and Mwaka [24], which found primary school children in a district of South Africa had very little awareness of nutrition. Similar to our finding, the study suggested that the lack of awareness might be attributed to a lack of emphasis within the formal school system. Many of the interviewed primary school children who had a conscious awareness of nutrition identified family members as their predominant source of nutrition information. This finding agrees with Scaglioni *et al*.’s [1] review which framed families as the primary unit of socialization hence a significant contributor to the nutrition behaviors formed in children. Many of the children confirmed that they were often involved in household grocery shopping and meal preparations at home and that may have been an avenue for the transfer of nutrition knowledge. About two-thirds of the interviewed homebased caregivers reported consciously coming across nutrition messages or nutrition-related messages. A study by Williams *et al*., [25]), indicated that the awareness of nutrition that a mother had did not necessarily translate into children having healthful diets. However, Zarnowiecki *et al*., [8] found a positive association between parental nutrition knowledge and young children’s nutrition knowledge.

Family members as a source of nutrition information was closely followed by lessons learned in class, indicating the important role that the school system plays in imparting nutrition knowledge in children especially during their formative years. A systematic review by Dudley, Cotton and Peralta [26] found that some primary school children became conscious of healthy eating behaviors through engagement in classroom lessons that were suitable for their stage of learning. Taking a close look at these two sources of information, i.e., family members and lessons learned in class, teachers who were interviewed suggested the possibility of bringing parents on board to ensure NE works in the children’s favor. Golley, Hendrie, Slater & Corsini, [27] showed that nutrition programs with direct parent involvement improved children’s nutritional status. Also, such interventions have positive associations with physical activity levels and healthy eating behaviors [28]. Many of the respondents in our study mentioned the vital role the Parent Teacher Association could have in developing a sustainable nutrition and physical education program within the primary school system. Given successes of earlier partnerships with the school authorities, parents could drive the NE agenda from home. Furthermore, they could initiate and sustain the discussion on nutrition within the basic school environment.

The eclectic sources of nutrition-related information may partly explain why nutrition was understood differently by different respondents. Most teachers reported gaining their understanding of nutrition from external sources. However, the information from these sources could only be considered as complementary as the kind of information provided may have been suited to the external source’s own program objectives and not necessarily targeted at informing to influence the nutrition behaviors of school age children. Some teachers also mentioned acquiring nutrition information from the internet. Teachers in this study could not mention the specific sources from which they obtained the nutrition information. This is crucial, as the information would be channeled into nutrition related activities in the school. Teachers actually admitted that they felt limited in their knowledge and how that could possibly affect their attitudes and behaviors towards the subject of nutrition. This phenomenon was also observed by Kupolati *et al*. [29], as well as Rapson, Conlon & Ali [13], who’s study participants indicated that their lack of formal training in nutrition made them feel inadequate at teaching and implementing nutrition education in the classroom setting. These diverse nutrition knowledge systems may have implications for the kind of knowledge system that is applied. The disparities in nutrition knowledge may result in different outcomes for nutrition which may not be easily observed because nutrition outcomes may take a while to manifest especially among children of school going age. All the school cooks in this study were mothers and therefore had benefitted from nutrition education given for prenatal and postnatal purposes during and after pregnancy. School cooks noted that the nutrition education received from the hospital was the main and sometimes only source of information applied to cooking in the basic school. While this information may have been useful for their personal consumption, its sufficiency for school going children at different ages is a relevant question.

Pertaining to the perceived barriers, teachers explained that there is no formal training for teaching nutrition and from the results, knowledge insufficiency was identified and less than half of the teachers interviewed felt confident to teach NE. Nutrition Education is often not taken seriously because it is “buried” in some other science course in primary school settings [30, 31]. Teachers admitted that because NE were not examined in the Basic Education Certificate Examination, teaching staff did not prioritize them. According to some respondents, teachers viewed NE as extra work that did not really count hence the neglect. With time, the focus of school authorities shifted to seemingly more important examinable subjects to the detriment of NE. It was also observed in our study that all the cooks indicated that their level of nutrition knowledge was sufficient for managing food at the school canteen. This pattern was interesting because school cooks often had the least education attainment. According to Spronk, Kullen, Burdon & O’Connor [32], the higher a person’s education qualification, the higher their knowledge on nutrition and appropriate dietary behaviors. A possible explanation for this observation among school cooks is that they may not have sufficient information to calibrate their actual extent of knowledge. It is also plausible that admitting their limitations may have implications for conducting their business in the school. Given all these reasons, it is therefore easy for school cooks to have an assuring posture and be seen to know what it takes to make the right choices as they conduct their food sales business.

Another perceived barrier we identified was the lack of resources for practical delivery and school-age children’s active participation in NE. Schneider & Theobald [33], exemplified in their study how organized and uniform nutrition learning kits developed for teachers can equip them with adequate and credible nutrition information to teach children nutrition. A similar approach may be crucial in environments where the NE policy is not known readily amongst key actors of the educational sector. Presently, there are no NE guidelines readily available, the third perceived barrier we identified, to even subject teachers; they may have to depend on their own nutrition knowledge to carry out nutrition related functions in the formal school system. This may be a faulty approach as scholarly work has indicated that teachers are more confident to teach nutrition when they have expert guidelines [33].

The lack of formal training for teachers in NE, lack of resources including models and kits for practical delivery and engagement, and the unavailability of official NE guidelines within the educational set up challenges nutrition within the school environment. The result is that nutrition and related activities are administered on an ad hoc basis. This is primarily because the only measurable outcome of nutrition observed in this study was the prevention of an outbreak of a disease associated with poor hygiene.

These findings should be interpreted with caution. Not all factors surrounding the acquisition of nutrition information was captured. Additional information on timing and actual quality of the nutrition information received from any of the sources was not assessed. Possible barriers to the inclusion of nutrition education in the mainstream primary school curriculum may have not been captured due to the subjective nature of FGDs and KIIs. Also, only four schools were used for the study, thus limiting the generalizability of the study findings in Ghana.

## Conclusion

The responses provided by stakeholders in this study has important implications for the design of interventions and policy development. The relevance of nutrition education had long been recognized by all the key stakeholders. However, the observable issues are related with the school system and not necessarily an absence of knowledge of NE’s importance. Decisions and policies formulated to benefit the entire school system should consider formally training teachers on NE and its dissemination across the stages of primary school, and provision of appropriate learning materials and kits. Lastly, NE directives can be designed to focus on creating a community where different types of contributions to nutrition are recognized and deliberately included in order to reinforce what children are taught about nutrition both at school and at home.

## Supporting information

S1 File Interview guides for key informant interviews and focus group discussions. (PDF)
