# Supplementary material for: Nutrition education incorporation into mainstream primary school curriculum in Ghana: Stakeholders’ sources of nutrition information and perceived barriers
Source: PLoS One. 2022 Jan 6;17(1):e0262359. doi: 10.1371/journal.pone.0262359 (PMC8735612; doi:10.1371/journal.pone.0262359)
Supplement: S1 File — (PDF) [file pone.0262359.s001.pdf]

Interview Guides for Key Informant Interviews and Focus Group Discussions

Key Informant In-depth Interview Guide for GES Officials

Key Informant In-depth Interview Identification Number: …………………………………..

Name of Facilitator/Researcher: …………………………………………………………….

Community/district: …………………………………………………………………………….

Gender of Interviewee: ....……………………………………………………………........

Date: …………………………………………………………………………………………….

Time started: ……………………………….. Time Ended: …....……………………………………

A) Introduction after informed consent (5 minutes)

Before proceeding, facilitator (research assistant) must read consent statement and ensure that the participant has signed or given a verbal consent to be interviewed.

1. Introduce yourself and any other team members present.
2. Inform him/her that you will switch on the audio recorder; make sure that the audio recorder is on.
3. Explain ground rules from the consent form. Say: "Thank you so much for agreeing to be interviewed. Please remember that your participation is voluntary and you can end the interview if you feel uncomfortable or if you want to stop the interview. Remember that all information shared during the interview will be anonymised and treated confidential."

Note for the facilitator/ research assistant: This in-depth interview will touch on four main areas: 1) GES nutrition and physical education (PE) policy and Standard 2) Incorporating nutrition education into PE curriculum and Policy 3) Involving Parents and Cooks to Teach Nutrition 4) GES' Position and Support for Nutrition Education. After exhausting each question item, indicate by ticking.

B) General information for purpose of analysis

This basic information is relevant for data analysis, so ensure that you spend some little time to take note of the interviewee's demographic details.

a. Could you tell me a little bit of information about yourself?
b. If you are married, how long have you been married? If you have any children, how many do you have and how many are in primary school?
c. How long have you been working as a GES official?

1.1 GES PE policy and Standard

	Tick here
a. What is the GES policy and standard for PE in the primary schools' curricula?	
b. Compared to the available policy and standard, what is the current state of physical education (PE) in our primary schools (class 1-6)?	
c. What does PE entail in the primary schools' curriculum?	
d. What is your perception of the PE program, do you think it has achieved its objectives.	
e. How does GES ensure that PE is enforced in every primary school?	
f. How are teachers allocated to teach PE? Are there specific PE teachers or it is the science teachers that teach PE?	

1.2 Incorporating nutrition education into PE curriculum and Policy

	Tick here
a. What is your perception? Should nutrition education (explain – providing students with the knowledge on the food groups, the need to eat from all the food groups and the relationship between nutrition and health therefore enhancing their skills and motivation to make healthy food choices, practice hygiene and food safety) be part of the PE curriculum? Is it important?	
b. How do you think providing nutrition education as part of the curriculum will be helpful to the child's development in terms of	
i. nutrition and health?	
ii. academic or education achievement?	
c. Is providing PE to the primary school students written into the GES school standards/job description for teachers? What about providing nutrition education?	
d. How will GES coordinate with the schools' principals and support to effect changes to the curriculum of PE to incorporate nutrition education, with available evidence that nutrition education is effective for nutrition KAP and improving nutrition status, ultimately academic achievement?	

1.3 Involving Homebased Caregivers and School Cooks to Teach Nutrition

	Tick here
Should parents be involved in teaching nutrition education to students in the primary schools? What effectiveness will their role play when they are involved in the lessons and activities?	
What is your opinion about the role of the school cooks in incorporating nutrition education into the school food supply/canteen?	
Do the school cooks need to have knowledge about nutrition education or nutrition?	

1.4 GES' Position and Support for Nutrition Education

	Tick here
a. Does GES encourage providing nutrition education to primary school students?	
b. Are there any funds from GES or government allocated to support provision of resources for nutrition education to primary school students, in terms of	
i. lesson/curriculum materials?	
ii. activities, videos, games, posters, and models?	

Closing: Thank the participants for their time and ideas, and express how helpful it has been for the facilitator(s) and the study.

Key Informant In-depth Interview Guide for School Principals

Key Informant In-depth Interview Identification Number: ……………………………………..

Name of Facilitator/Researcher: ……………………………………………………………….

Community/district: …………………………………………………………………………….

Gender of Interviewee: ....……………………………………………………………….......

Date: …………………………………………………………………………………………….

Time started: …………………………….. Time Ended: …...…………………………………

A) Introduction after informed consent (5 minutes)

Before proceeding, facilitator (research assistant) must read consent statement and ensure that the participant has signed or given a verbal consent to be interviewed.

1. Introduce yourself and any other team members present.
2. Inform him/her that you will switch on the voice recorder; make sure that the voice recorder is on.
3. Explain ground rules from the consent form. Say: "Thank you so much for agreeing to be interviewed. Please remember that your participation is voluntary and you can end the interview if you feel uncomfortable or if you want to stop the interview. Remember that all information shared during the interview will be anonymised and treated confidential."

Note for the facilitator/ research assistant: This in-depth interview will touch on four main areas: 1) GES nutrition and PE policy and Standard 2) Incorporating nutrition education into PE curriculum and Policy 3) Involving Parents and Cooks to Teach Nutrition 4) Head-teacher's and GES' Support for Nutrition Education. After exhausting each question item, indicate by ticking.

B) General information for purpose of analysis

This basic information is relevant for data analysis, so ensure that you spend some little time to take note of the interviewee's demographic details.

a. Could you tell me a little bit of information about yourself?
b. If you are married, how long have you been married? If you have any children, how many do you have and how many are in primary school?
c. How long have you been working as a school principal?

1.1 GES PE policy and Standard

	Tick here
What is the GES policy and standard for PE in the primary schools' curricula?	
What does PE entail in your primary schools' (class 1-6) curriculum?	
How do you ensure that PE is enforced in your primary school?	
What is your perception of the PE program, do you think it has achieved its objectives.	
How are teachers allocated to teach PE? Are there specific PE teachers or it is the science teachers that teach PE?	
How many PE teachers do you have currently? Is there a PE teacher for each class (1-6)?	

1.2 Incorporating nutrition education into PE curriculum and Policy

	Tick here
a. Is nutrition education part of your primary students' curriculum?	
b. What is your perception? Should nutrition education (explain – providing students with the knowledge on the food groups, the need to eat from all the food groups and the relationship between nutrition and health therefore enhancing their skills and motivation to make healthy food choices, practice hygiene and food safety) be part of the PE curriculum? Is it important or valued?	
c. Whose role is it to provide nutrition education to your primary students?	
d. How do you think providing nutrition education as part of the curriculum will be helpful to the child's development in terms of	
i. nutrition and health?	
ii. academic or education achievement?	
e. Is providing PE to the primary school students written into the GES school standards/job description for teachers? What about providing nutrition education? Do teachers provide nutrition education to your primary students?	
f. How will school headteachers coordinate with the GES and support to effect changes to the curriculum of PE to incorporate nutrition education, with available evidence that nutrition education is effective for nutrition KAP and improving nutrition status, ultimately academic achievement?	

1.3 Involving Homebased Caregivers and School Cooks to Teach Nutrition

	Tick here
Should parents be involved in teaching nutrition education to students in the primary schools? What effectiveness will their role play when they are involved in the lessons and activities?	
What is your opinion about the role of the school cooks in incorporating nutrition education into the school food supply/canteen? (Participation, foods available)	
Do the school cooks need to have knowledge about nutrition education or nutrition?	

1.4 Headteacher and GES' Support for Nutrition Education

	Tick here
a. Do you as the school's headteacher encourage providing nutrition education to primary school students?	
b. Are there any funds from GES, government or school board allocated to support provision of resources for nutrition education to primary school students, in terms of,	
i. lesson/curriculum materials?	
ii. activities, videos, games, posters, and models?	

Closing: Thank the participants for their time and ideas, and express how helpful it has been for the facilitators.

Key Informant In-depth Interview Guide for School Teachers

Key Informant In-depth Interview Identification Number: ……………………………………..

Name of Facilitator/Researcher: ………………………………………………………………….

Community/district: …………………………………………………………………………….

Gender of Interviewee: ....…………………………………………………………………........

Date: …………………………………………………………………………………………….

Time started: ……………………………. Time Ended: ……....………………………………

A) Introduction after informed consent (5 minutes)

Before proceeding, facilitator (research assistant) must read consent statement and ensure that the participant has signed or given a verbal consent to be interviewed.

1. Introduce yourself and any other team members present.
2. Inform him/her that you will switch on the voice recorder; make sure that the voice recorder is on.
3. Explain ground rules from the consent form. Say: "Thank you so much for agreeing to be interviewed. Please remember that your participation is voluntary and you can end the interview if you feel uncomfortable or if you want to stop the interview. Remember that all information shared during the interview will be anonymised and treated confidential."

Note for the facilitator/ research assistant: This in-depth interview will touch on six main areas: A) Teachers' Knowledge and perception of PE programs 2) Incorporating Nutrition Education in School Curriculum 3) Teacher's Experience with Teaching Nutrition 4) School Projects Related to Nutrition 5) Involving Parents and Cooks to Teach Nutrition 6) Head-teacher's and GES' Support for Nutrition Education. After exhausting each question item, indicate by ticking.

B) General information for purpose of analysis

This basic information is relevant for data analysis, so ensure that you spend some little time to take note of the interviewee's demographic details.

a. Could you tell me a little bit of information about yourself?
b. If you are married, how long have you been married? If you have any children, how many do you have and how many are in primary school?
c. How long have you been working as a teacher?

1.1 Teachers' Knowledge and perception of PE programs

	Tick here
a. How do you ensure that PE is enforced in your primary school?	
b. What is your perception of the PE program, do you think it has achieved its objectives.	
c. How are teachers allocated to teach PE? Are there specific PE teachers or it is the science teachers that teach PE?	
d. How many PE teachers does your school have currently? Is there a PE teacher for each class (1-6)?	
e. Are you involved in the activities of the PE program?	
f. Is providing PE to the primary school students written into the GES school standards/job description for teachers? What about providing nutrition education?	

1.2 Incorporating Nutrition Education in School Curriculum

	Tick here
a. Is nutrition education part of your primary students' curriculum?	
b. How do you think providing nutrition education as part of the curriculum will be helpful to the child's development in terms of…	
i. nutrition and health?	
ii. academic or education achievement?	
c. What is your perception? Should nutrition education (explain – providing students with the knowledge on the food groups, the need to eat from all the food groups and the relationship between nutrition and health therefore enhancing their skills and motivation to make healthy food choices, practice hygiene and food safety) be part of the PE curriculum? Is it important or valued?	
d. Whose role is it to provide nutrition education to your primary students?	

1.3 Teacher's Experience with Teaching Nutrition

	Tick here
a. During this school year (2018), have you taught lessons on nutrition to your students?	
b. What are your sources of information on nutrition issues? Are they adequate?	
c. What are your views about? a). Students" value of nutrition? b). Do they practice what is taught?	
d. Which instructional materials do you use for teaching nutrition in the school? Are they adequate?	
e. What practical skills do you offer that contribute to nutrition practice?	
f. What challenges and barriers do you face in the teaching of nutrition? PROBE for teaching resources: books, teaching aids and materials; time allocated in the time-table; interest among the pupils, follow formal syllabus and lesson plans?	
g. Do you feel the present curriculum adequately covers nutrition? If NOT, what aspects are not included and you feel are important?	

1.4 School Projects Related to Nutrition

	Tick here
a. Do the students in this school participate actively in projects initiated in the school (school feeding program) among others available?	
b. Do you think the participation in school projects and clubs has led to improved food, nutrition and environmental knowledge among the pupils? If yes, how?	
c. Which projects in particular have been very instrumental in the above?	
d. Please comment on the students' interest and commitment to these project activities.	

1.5 Involving Homebased Caregivers and School Cooks to Teach Nutrition

	Tick here
a. In what ways have parents contributed to the nutrition knowledge and practices of the pupils? In what other ways can parents be involved in nutrition education in order to improve the nutrition knowledge, attitudes and practices of the pupils?	
b. What is the parents' level of interest and commitment to these project activities?	
c. In what ways have parents and community perceptions, in your view influenced the nutrition attitudes of the pupils?	
d. Should parents be involved in teaching nutrition education to students in the primary schools? What effectiveness will their role play when they are involved in the lessons and activities?	
e. What is your opinion about the role of the school cooks in incorporating nutrition education into the school food supply/canteen? (participation, foods available)	
f. Do the foodservice staff or cooks need to have knowledge about nutrition education or nutrition?	

1.6 Head-teacher's and GES' Support for Nutrition Education

	Tick here
a. Does the school's head-teacher encourage providing nutrition education to primary school students?	
b. Are there any funds from GES, government or school board allocated to support provision of resources for nutrition education to primary school students, in terms of	
i. lesson/curriculum materials?	
ii. activities, videos, games, posters, and models?	

Closing: Thank the participants for their time and ideas, and express how helpful it has been for the facilitators.

Key Informant In-depth Interview Guide for School Cooks

Key Informant In-depth Interview Identification Number: ……………………………………..

Name of Facilitator/Researcher: …………………………………………………………………….

Community/district: ……………………………………………………………………………….

Gender of Interviewee: ....………………………………………………………………………........

Date: …………………………………………………………………………………………………….

Time started: …………………………….. Time Ended: …....………………………………….

A) Introduction after informed consent (5 minutes)

Before proceeding, facilitator (research assistant) must read consent statement and ensure that the participant has signed or given a verbal consent to be interviewed.

1. Introduce yourself and any other team members present.
2. Inform him/her that you will switch on the voice recorder; make sure that the voice recorder is on.
3. Explain ground rules from the consent form. Say: "Thank you so much for agreeing to be interviewed. Please remember that your participation is voluntary and you can end the interview if you feel uncomfortable or if you want to stop the interview. Remember that all information shared during the interview will be anonymised and treated confidential."

Note for the facilitator/ research assistant: This in-depth interview will touch on six main areas: four main areas: 1) nutrition knowledge 2) school feeding program 3) physical education in schools 4) Involving Parents and Cooks to Teach Nutrition. After exhausting each question item, indicate by ticking.

B) General information for purpose of analysis

This basic information is relevant for data analysis, so ensure that you spend some little time to take note of the interviewee's demographic details.

a. Could you tell me a little bit of information about yourself?
b. If you are married, how long have you been married? If you have any children, how many do you have and how many are in primary school?
c. How long have you been working as a teacher?

1.1 Nutrition knowledge

	Tick Here
a. What is nutrition? What is its value to you, the school community as a whole?	
b. What are the sources of nutrition knowledge in this community? PROBE for:	
i. relatives/friends	
ii. from school	
iii. community	
iv. from your children	
c. Have you acquired any new information on health and nutrition from any health agency this year?	
d. If so, what information have you acquired? How useful is this information?	

1.2 School feeding program

	Tick here
Do you receive any in-service training on how to ensure that the food you cook and serve has all the five food groups, including fruits and vegetables?	
Have you learnt any storage and preservation techniques for your cooking?	
Do you think children are getting sufficient food and nutrients through the school lunch provided by your organization? Or it can be improved?	

1.3 Physical education in schools

	Tick here
What is your view about the PE program?	
In your view, what else could be done to improve on the activities?	

1.4 Involving Homebased Caregivers and School Cooks to Teach Nutrition

	Tick here
Should parents be involved in teaching nutrition education to students in the primary schools? What effectiveness will their role play when they are involved in the lessons and activities?	
What is your opinion about the role of the foodservice staff or cooks in incorporating nutrition education into the school food supply/canteen? (participation, foods available)	
Do the foodservice staff or cooks need to have knowledge about nutrition education or nutrition and provide nutrition education to primary students?	

Closing: Thank the participants for their time and ideas, and express how helpful it has been for the facilitators.

Participatory Focus Group Discussion Guide for Homebased Caregivers

PFGD Identification Number: ……………………………………………………………………………

Name of Facilitator/Researcher: …………………………………………………………………….

Community/district: ………………………………………………………………………………….

Date: ……………………………………………………………………………………………….

Time started: ……………………………….. Time Ended: ……...………………………………….

A) Introduction after informed consent (5 minutes)

Before proceeding, facilitator (research assistant) must read consent statement and ensure that all participants have signed or given a verbal consent to be part of the PFG.

1. Introduce yourself and any other team members present.
2. Inform them that you will switch on the voice recorder; make sure that the voice recorder is on.
3. Explain ground rules from the consent form. Say: "Thank you so much for agreeing to participate in this PFGD. Please remember that your participation is voluntary and you can leave if you feel uncomfortable or you want to stop the discussion. Remember that all information shared during the discussions will be anonymised and treated confidential." Only one person should speak at a time.
4. Ask participants to suggest other ground rules that will help them feel comfortable sharing their ideas during the group discussion.
5. If the participants have not made any ground rules about phone etiquettes, ask them about it and help them understand that their phones should be muted during the PFGD process.

Note for the facilitator/ research assistant: This PFGD will touch on four main areas: 1) nutrition knowledge 2) school feeding program 3) physical education in schools 4) and challenges at home regarding the provision of well-balanced nutrition and meal. After exhausting each question item, indicate by ticking.

1.1 Nutrition Knowledge

	Tick here
a. Ask the participants to share their understanding of what nutrition is.	
b. Ask them to tell you the value of nutrition to	
I. Them	
II. their family	
III. their community	
c. Ask them to share with the sources of nutrition knowledge in their community	
Probe for the following sources;	
I. Relatives/friends	
II. Community	
III. From school	
IV. From child(ren)	
d. Ask them further whether they have acquired any new information on health and nutrition from their child(ren)	
I. If any of them have, ask them to share the information with and share with you how useful the information is.	
e. Explore their thoughts on whether their children are being equipped with adequate information on health and nutrition at school.	
I. Find out from them if they have learnt any storage and preservation techniques through transfer of knowledge from their child(ren) from school to home.	

1.2 School Feeding Program

	Tick here
a. Find out from them whether they are aware of the school feeding program in their child(ren) school.	
Probe about the following	
I. Who funds the school feeding program	
II. Their attitudes towards the school feeding program	
III. Their contributions to the school feeding program	
b. Explore their thoughts on whether children are getting sufficient food and nutrients through the school lunch program by the school feeding program	
c. Find out whether they like the food their children are served in the school.	

1.3 Nutrition in Physical Education

	Tick here
a. Explore their views on physical education program (it's commonly known as PE) in schools. Find out whether it's a good program and how good it is.	
b. Explore how the PE program has been beneficial to their children and whether it has made contributions to their nutrition education.	
c. Ask them to share with you the ways in which the PE program has contributed/helped their child(ren)'s nutrition education.	
d. PROBE: their views on the nutrition education activities undertaken by the school where their child(ren) goes	
e. Find out from them the ways by which nutrition in PE activities can be improved in the schools	

Closing: Thank the participants for their time and ideas, and express how helpful it has been for the facilitators.

Participatory Focus Group Discussion Guide for School-Age Children

PFGD Identification Number: …………………...

School: ....…………………………………………………………………………………………..

Name of Facilitator/Researcher: …………………………………………………………………….

Community/district: ……………………………………………………………………………….

Date: ……………………………………………………………………………………………….

Time started: …………………………….. Time Ended: ……...…………………………………

A) Introduction after assent form (5 minutes)

Before proceeding, facilitator (research assistant) must read assent form statement and ensure that all parents/guardians have assented for their children to participate in PFGD.

1. Introduce yourself and any other team members present.
2. Inform them that you will switch on the voice recorder; make sure that the voice recorder is on.
3. Explain ground rules from the consent form. Say: "Thank you so much for agreeing to participate in this PFGD. Please remember that your participation is voluntary and you can leave if you feel uncomfortable or you want to stop the discussion. Remember that all information shared during the discussions will be anonymised and treated confidential." Only one person should speak at a time.
4. Ask participants to suggest other ground rules that will help them feel comfortable sharing their ideas during the group discussion.
5. If the participants have not made any ground rules about phone etiquettes, ask them about it and help them understand that their phones should be muted during the PFGD process.

Note for the facilitator/ research assistant: This PFGD will touch on two main areas: 1) nutrition knowledge 2) basic food and water hygiene knowledge. The first part is task base, which requires that you present food items to pupils and explore their knowledge on them. After exhausting each question item, indicate by ticking.

1.1 Nutrition Knowledge and Sources of Nutrition Information

1. What is nutrition?

2. What constitutes a healthy well-balanced meal for you and your family?

3. How is nutrition related to health and your academic performance?

4. Have you acquired any new information on health and nutrition from your teacher?

5. If so, what information have you acquired? How useful is this information?

6. Do you think that you are being equipped with adequate information on health and nutrition at the school?

7. From where do you receive information about nutrition issues? (PROBE WITH OPTIONS BELOW).

1= I do not receive any nutrition information [ ]

2= Text books [ ]

3= Newspapers /pamphlets [ ]

4= Family members [ ]

5= Friends (parents) [ ]

6= Television [ ]

7= Radio [ ]

8= From lessons taught in class [ ]

9= Physical Education program activities in the school [ ]

10= Physical Education program activities in the community [ ]

11= Any other (specify)………………………………………………………

8. Which of the sources mentioned above provide you with the most information? Rank in order of their importance)

1. ………………………………………………………………………………

2. ………………………………………………………………………………

3. ………………………………………………………………………………

9. Which of these three sources of nutrition information do you like most? (List in order of their liking) 1. …………………………………………………………………………….....

2. ………………………………………………………………………………

3. ………………………………………………………………………………

Focus group discussions members' demographic data form: This form will be useful for socio-demographic data capture

School name__________________Location___________________________Date______________

Number	Name	Gender	Age	Marital status	Education level	Name of group	Sign
